# Acute Myocardial Infarction Caused by Pulmonary Vein Stump Thrombosis after Thoracoscopic Left Upper Lobectomy

**DOI:** 10.70352/scrj.cr.24-0003

**Published:** 2025-01-30

**Authors:** Takahito Fukushima, Masaaki Nagano, Yue Cong, Tatsuki Furusawa, Akihito Saito, Shun Minatsuki, Satoshi Kodera, Norihiko Takeda, Masaaki Sato

**Affiliations:** 1 Department of Thoracic Surgery, Graduate School of Medicine, The University of Tokyo, Tokyo, Japan; 2 Department of Thoracic Surgery, National Center for Global Health and Medicine, Tokyo, Japan; 3 Department of Cardiovascular Medicine, Graduate School of Medicine, The University of Tokyo, Tokyo, Japan

**Keywords:** lung cancer, left upper lobectomy, pulmonary vein stump thrombosis, acute myocardial infarction

## Abstract

**INTRODUCTION:**

Pulmonary vein stump thrombosis can sometimes occur at the pulmonary vein stump after lung surgery, possibly causing systemic infarction. Here, we report a rare case of acute myocardial infarction (AMI) caused by pulmonary vein stump thrombosis after the left upper lobectomy.

**CASE PRESENTATION:**

A 43-year-old male patient with a nodule in the left lingular segment was referred to our hospital. A bronchoscopic biopsy performed at the previous hospital was negative for malignancy; however, the nodule was highly suspicious of primary lung cancer. Therefore, we decided to perform a thoracoscopic lung resection for a definite diagnosis and treatment. Lingular segmentectomy was performed to diagnose the nodule, and a rapid pathological diagnosis confirmed that the nodule was an adenocarcinoma. Subsequently, a left upper lobectomy and systemic lymph node dissection were performed. The left lingular and superior segmental veins were separately dissected using a stapler. The day after the operation, the patient suddenly developed cardiac arrest. Cardiopulmonary resuscitation and venoarterial extracorporeal membrane oxygenation were immediately initiated. After the return of spontaneous circulation was obtained, contrast computed tomography was performed, which suggested thrombosis of the pulmonary vein stump without any signs of brain hemorrhage or infarction. As intermittent ventricular fibrillation persisted, the patient underwent coronary angiography and was diagnosed with AMI due to pulmonary vein stump thrombosis. The thrombosis of the coronary artery was removed using percutaneous coronary intervention. The patient recovered gradually after the intervention and was discharged 2 weeks later from the intensive care unit. One month after rehabilitation for higher brain dysfunction, the patient was discharged from our hospital without any sequelae and received adjuvant chemotherapy for lung cancer.

**CONCLUSIONS:**

We encountered a case of AMI caused by pulmonary vein stump infarction after the left upper lobectomy. Given that this complication is rare but lethal, clinicians should consider it and take great care of the residual length of the pulmonary vein stump to prevent thrombosis.

## Abbreviations

AMI
: acute myocardial infarction
CT
: computed tomography
ECMO
: extracorporeal membrane oxygenation
IABP
: intra-aortic balloon pumping
LUL
: left upper lobectomy
PVST
: pulmonary vein stump thrombosis
RCA
: right coronary artery

## INTRODUCTION

Pulmonary vein stump thrombosis (PVST) is an uncommon postoperative in situ thrombosis occurring at the stump of the pulmonary vein after lung resection, especially left upper lobectomy (LUL). A prospective multi-institutional study in Japan detected PVST in 21% of LUL cases and 1.6% of other types of lobectomies.^1)^ PVST can cause systemic infarction, with cerebral infarction being the most common.^1,2)^ Infarctions in other organs caused by PVST include renal,^3)^ splenic,^4)^ and spinal cord infarctions.^5)^ However, there are no reports of acute myocardial infarction (AMI) caused by PVST. Here, we report a rare case of LUL and sudden cardiac arrest due to occlusion of the right coronary artery (RCA) by the PVST on the day after surgery.

## CASE PRESENTATION

**Fig. 1:**
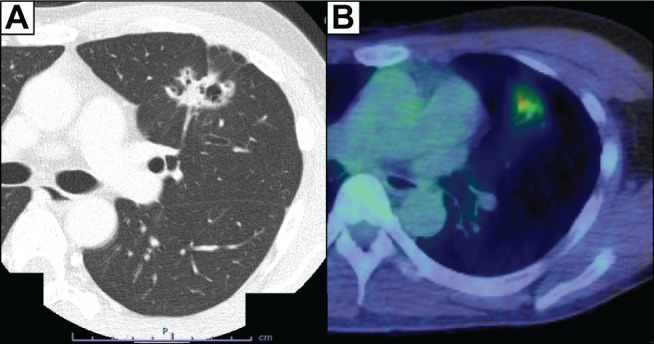
Preoperative CT. Contrast CT showed the pure-solid nodule (diameter: 3.3 cm) in the left lingular segment (**A**), and PET-CT showed fluorodeoxyglucose uptake, with an SUV max of 3.7 for the same nodule (**B**). PET, positron emission tomography

**Fig. 2:**
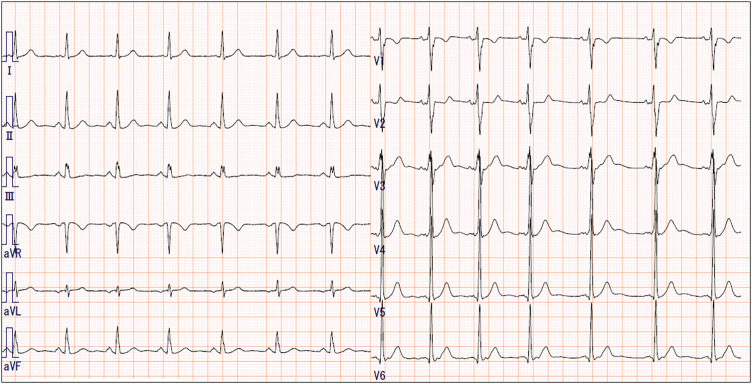
Preoperative electrocardiogram. The electrocardiogram before surgery showed a Wolff-Parkinson-White pattern.

The patient was a 43-year-old male. An abnormal shadow is detected in the left middle lung field on a health check-up radiograph. Contrast computed tomography (CT) showed a pure-solid nodule with spiculation and a size of 3.3 cm in diameter in the left lingular segment (**Fig. 1A**). No apparent coronary artery calcification was detected on the contrast-enhanced CT. A transbronchial biopsy of the nodule via flexible bronchoscopy was performed at the previous hospital, and it showed that the biopsy was negative for malignancy. However, the CT images strongly suggested primary lung cancer; thus, the patient was referred to our hospital for further examination 2 months later after treatment for pneumothorax and pneumonia caused by bronchoscopy. Fluorodeoxyglucose positron emission tomography revealed abnormal accumulation in the nodule in the left upper lobe (**Fig. 1B**), and the nodule size remained unchanged. The physical examination indicated the following: height of 176 cm, weight of 63 kg, and body mass index of 20.3 kg/m^2^. He had no risk factors for coronary artery disease including hypertension, dyslipidemia, diabetes, family history, smoking, and obesity. The electrocardiogram showed a Wolff-Parkinson-White pattern (**Fig. 2**), but he had never had any episodes of tachycardia. Although we offered the option of re-biopsy of the nodule via bronchoscopy, the patient refused this option and wanted to undergo surgery for a definite diagnosis and treatment.

**Fig. 3:**
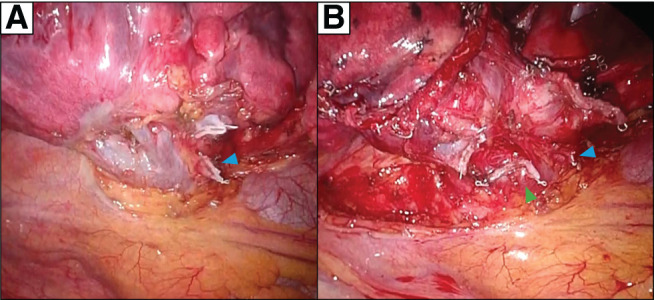
Intraoperative findings. The pulmonary vein of the lingular segment is resected using a stapler (**A**), and the vein of the superior segment is resected separately (**B**). Arrowhead (blue), pulmonary vein stump of the lingular segment; arrowhead (green), pulmonary vein stump of the superior segment.

The patient underwent thoracoscopic surgery. First, a left lingular segmentectomy was performed because the diagnosis of the nodule had not yet been established. The nodule was found to be an adenocarcinoma by intraoperative frozen section; thus, we performed LUL and systemic lymph node dissection. The left lingular and superior segmental veins were dissected separately using a stapler (**Fig. 3**). The operation time was 2 h and 16 min, and intraoperative blood loss was minimal. The patient was extubated in the operating room and returned to the regular ward in stable condition.

**Fig. 4:**
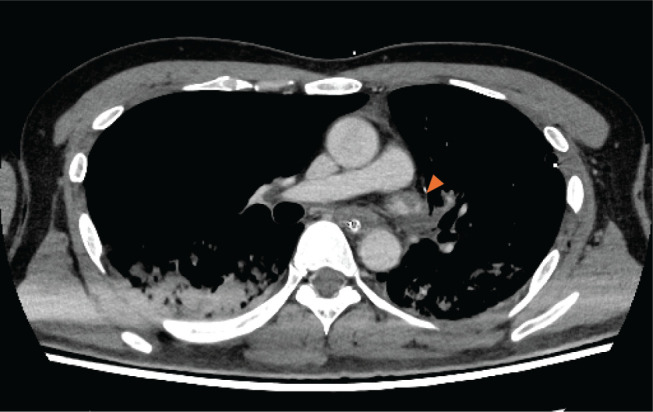
Thromboses on CT. Contrast CT immediately after the return of spontaneous circulation suggested thrombosis of the pulmonary vein stump. Arrowhead, pulmonary vein stump

**Fig. 5:**
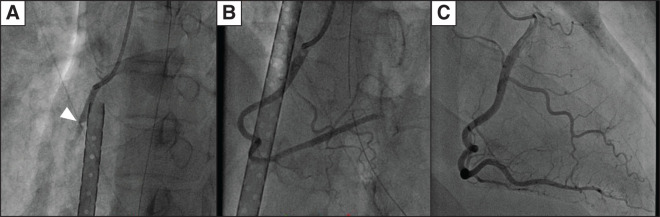
Coronary angiography. Coronary angiography shows occlusion of the right coronary artery (arrowhead) (**A**). Most blood clots were removed by percutaneous coronary intervention, although some remained in the peripheral coronary artery (**B**). Follow-up coronary angiography before discharge shows no apparent stenosis in the coronary arteries (**C**).

The following morning, no abnormalities were found in postoperative blood or radiological examinations. The patient could wake up and walk smoothly without any symptoms, and the cardiac monitor was removed at 10 a.m. His consciousness was clear until 11:45, although he complained of slight chest pain and dizziness. However, the patient experienced a cardiac arrest at approximately noon. Electrocardiography revealed ventricular fibrillation and cardiopulmonary resuscitation was immediately initiated. As the return of spontaneous circulation was not achieved immediately, veno-arterial extracorporeal membrane oxygenation (ECMO) was introduced. After spontaneous circulation was secured, a contrast CT scan was performed, which showed that thrombosis of the pulmonary vein stump was highly suspected (**Fig. 4**). No brain hemorrhage or infarction was observed on CT. Furthermore, intermittent ventricular fibrillation persisted, and an ultrasound cardiogram showed an ejection fraction of <30% with diffuse hypokinesis, especially in the anterior septum. Therefore, emergent coronary angiography was performed by cardiologists. A thrombotic occlusion in RCA #1 was found (**Fig. 5A**), and we determined that the cause of cardiac arrest was AMI caused by a coronary embolism from the PVST. Percutaneous coronary intervention, including thrombus aspiration, excimer laser coronary angioplasty, and perfusion balloon dilatation, was performed with intra-aortic balloon pumping (IABP) support. Although some blood clots remained on the peripheral side of the RCA, treatment was completed with coronary flow improvement (**Fig. 5B**). The patient was admitted to the intensive care unit with the extracorporeal membrane of ECMO and IABP.

His cardiac function gradually recovered, and ECMO was discontinued 3 days after surgery. The IABP was also removed 6 days after the surgery with stable hemodynamics. Anticoagulant treatment with heparin was started immediately after the intervention, and CT performed 4 days after surgery confirmed that the pulmonary vein thrombosis had disappeared. The patient was extubated 10 days after surgery and discharged from the intensive care unit.

Although head CT and MRI showed no apparent findings, higher brain dysfunction, including disturbances in attention and memory impairment, persisted for some time. After 1 month of rehabilitation for dysfunction, the patient was discharged without sequelae. No apparent stenosis was detected in the coronary arteries on follow-up coronary angiography before discharge (**Fig. 5C**). Direct oral anticoagulant therapy was continued after discharge.

The pathology of the lung nodule was adenocarcinoma with pT2aN2M0 p-stage IIIA, and adjuvant chemotherapy was initiated 2 months after the operation.

## DISCUSSION

We encountered a rare case of AMI due to occlusion of the RCA by a PVST after LUL. The thrombosis of the RCA was immediately removed by percutaneous cardiac intervention, and the patient was discharged without any sequelae 1 month after surgery.

To the best of our knowledge, this is the first study to demonstrate that PVST causes AMI and cardiac arrest after lung surgery. Previous studies have cautioned that PVST might cause systemic infarctions, including renal,^3)^ splenic,^4)^ and spinal cord infarctions.^5)^ It is noted that cerebral infarction is observed in 1%–4% of patients who underwent lung cancer surgery and may be caused by PVST in some patients.^1,2,6)^ However, no cases of coronary embolism caused by PVST after lung surgery have been reported. A systematic review showed that coronary embolism occurred primarily in patients with infective endocarditis, arterial fibrillation, and prosthetic heart valve thrombosis and that the left anterior descending coronary artery was the most frequently affected (45.3%), followed by the right (15.3%) and left circumflex (14.7%) coronary arteries.^7)^ This complication might be rare but can lead to a fatal course; therefore, it is necessary to carefully check the patient’s condition after the operation by monitoring the electrocardiogram. A multicenter observational study demonstrated that half of the PVSTs were diagnosed within 2 days after surgery, indicating electrocardiography should be performed at least a few days after surgery.^8)^

**Fig. 6:**
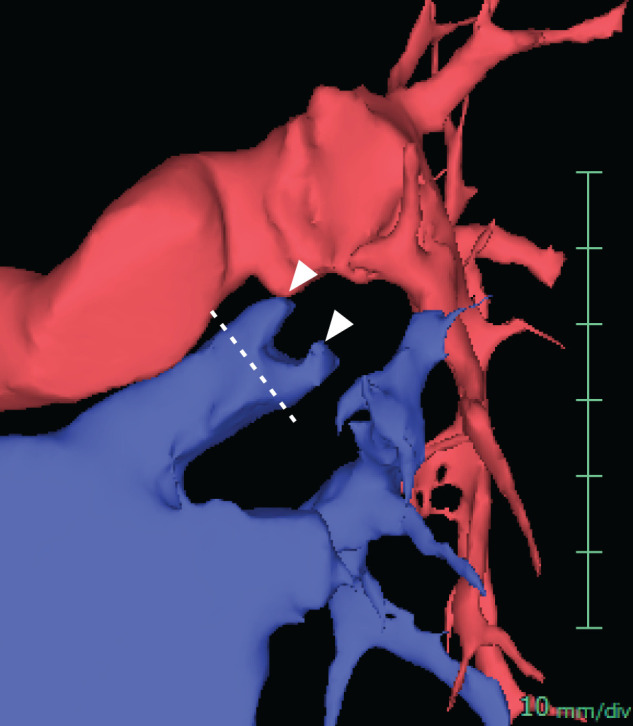
3D image of the pulmonary vein. Three-dimensional reconstruction of the pulmonary vein and artery showed that the pulmonary vein stump in this case (arrowheads) was longer than that in the usual left upper lobectomy (dotted line).

The longer-than-usual residual vein stump might be the main reason that our patient had early postoperative PVST formation. In 2013, Ohtaka et al. first reported that a long pulmonary vein stump after lung resection is associated with frequent PVST on contrast CT.^9)^ A few prospective multicenter observational studies have shown that left-sided lung resections, especially LUL, are a risk factor for PVST compared to right-sided lung resection because the remaining length of the pulmonary vein stump after LUL is significantly longer than that after other types of lobectomy.^1,8)^ Umehara et al. analyzed the mechanism of thrombus development after LUL. They suggested that blood flow stagnation or turbulence, vascular endothelial cell damage, and hypercoagulability near the long pulmonary vein stump might cause thrombus formation.^10)^ In our case, we stapled the pulmonary veins of the left superior and lingular segments separately because we first performed left lingular segmentectomy for a definite diagnosis, resulting in a longer vein stump than the usual resection line of the left superior pulmonary vein for the LUL (**Fig. 6**). Several papers suggest that thread ligation at the pericardial reflection could prevent PVST after lobectomy.^11,12)^ If we had used this method in this case, the shortened length of the remaining pulmonary vein stump could have prevented PVST and lethal complications. Recently, a multicenter randomized trial has shown better results of segmentectomy than lobectomy for non-small cell lung cancer with a tumor size of 2 cm or less.^13)^ However, the study also showed that some patients who underwent segmentectomy had to switch to lobectomy because of lymph node metastasis or insufficient surgical margins. Since these cases may require separate resection of the segmental pulmonary vein, surgical techniques that can make the residual length of the pulmonary vein stump as short as possible should be considered.

There are limitations to preventing the occurrence of PVST and its complications using surgical techniques alone. Anticoagulant therapy was reported beneficial for thrombus resolution when PVST was detected on contrast CT.^6,8,9)^ By contrast, the use of anticoagulant drugs immediately after surgery would not be preferable, considering the risk of hemorrhage. Furthermore, a meta-analysis showed that anticoagulant treatment did not improve the prognosis of lung cancer, although lung cancer patients with thrombosis had significantly worse overall survival than those without thrombosis.^14)^ In summary, the criteria for anticoagulation therapy remain unclear: when should the therapy be started or stopped? Which patients are eligible for treatment? Although a recent report indicated that the CHA2DS2-VASc score correlated with the formation of PVST,^15)^ the score was zero in our case. Therefore, multicenter prospective studies examining patients with lung cancer who can benefit from anticoagulant therapy to prevent PVST after lung surgery are required in the near future. In addition, how long anticoagulant treatment should be continued for the applicable patients needs to be elucidated in large clinical studies.

## CONCLUSIONS

Here, we report a rare case of AMI caused by PVST after lung surgery. This complication is rare but fatal; therefore, it should be considered, especially after LUL. In addition, the residual length of the pulmonary vein stump should be taken care of during surgery to prevent thrombosis.

## ACKNOWLEDGMENTS

We would like to thank Editage (www.editage.jp) for English language editing.

## DECLARATIONS

### Funding

This study received no funding.

### Authors’ contributions

The conception of the work: TF, MN, and YC

The acquisition, analysis, or interpretation of data for the work: TF, AS, SM, SK, NT, and MS

Drafting the work: TF, MN, YC, and AS

Revising it critically for important intellectual content: TF, SM, SK, NT, and MS

Final approval of the version to be published: all authors

Agreeing to be held accountable for all aspects of the work: all authors

### Availability of data and materials

The datasets supporting the conclusions of this article are included within the article.

### Ethics approval and consent to participate

This study was approved by the ethics committee of the University of Tokyo Hospital (clinical pilot study no. 2406).

### Consent for publication

Consent to publish this article has been obtained from the patient.

### Competing interests

The authors declare no conflicts of interest.
